# Quadriceps femoris spasticity in children with cerebral palsy: measurement with the pendulum test and relationship with gait abnormalities

**DOI:** 10.1186/1743-0003-11-166

**Published:** 2014-12-16

**Authors:** Andrzej Szopa, Małgorzata Domagalska–Szopa, Zenon Kidoń, Małgorzata Syczewska

**Affiliations:** School of Health Sciences, Medical University of Silesia, Katowice, Poland; Institute of Electronics, Silesian University of Technology, Gliwice, Poland; Paediatric Rehabilitation Department, The Children’s Memorial Health Institute, Warszawa, Poland

**Keywords:** Cerebral palsy, Clinical measurement of spasticity, Pendulum test, Gait analysis, Gillette gait index

## Abstract

**Background:**

Development of a reliable and objective test of spasticity is important for assessment and treatment of children with cerebral palsy. The pendulum test has been reported to yield reliable measurements of spasticity and to be sensitive to variations in spasticity in these children. However, the relationship between the pendulum test scores and other objective measures of spasticity has not been studied. The present study aimed to assess the effectiveness of an accelerometer-based pendulum test as a measurement of spasticity in CP, and to explore the correlation between the measurements of this test and the global index of deviation from normal gait in in children with cerebral palsy.

**Methods:**

We studied thirty-six children with cerebral palsy, including 18 with spastic hemiplegia and 18 with spastic diplegia, and a group of 18 typically-developing children. Knee extensor spasticity was assessed bilaterally using the accelerometer-based pendulum test and three-dimensional gait analysis. The Gillette Gait Index was calculated from the results of the gait analysis.

**Results:**

The data from the accelerometer-based pendulum test could be used to distinguish between able-bodied children and children with cerebral palsy. Additionally, two of the measurements, first swing excursion and relaxation index, could be used to differentiate the degree of knee extensor spasticity in the children with cerebral palsy. Only a few moderate correlations were found between the Gillette Gait Index and the pendulum test data.

**Conclusions:**

This study demonstrates that the pendulum test can be used to discriminate between typically developing children and children with CP, as well as between various degrees of spasticity, such as spastic hemiplegia and spastic diplegia, in the knee extensor muscle of children with CP. Deviations from normal gait in children with CP were not correlated with the results of the pendulum test.

**Electronic supplementary material:**

The online version of this article (doi:10.1186/1743-0003-11-166) contains supplementary material, which is available to authorized users.

## Background

Spasticity is one of the most common impairments in children with cerebral palsy (CP) [1]. Developing an objective measurement of spasticity has been a major goal for clinical researchers for many years, especially since the introduction of “spasticity management” [2].

Various clinical scales are used to assess spasticity, such as the (Modified) Ashworth Scale (MAS) [3], the (Modified) Tardieu Scale (MTS) [4] and the Dynamic Evaluation of Range of Motion (DAROM) scale [5, 6]. Only the MTS and the DAROM are based on Lance’s definition of spasticity [7]. All three scales [6–9] are widely used measures of spasticity and the response of patients to CP treatment, but the intrarater and interrater reliability of the MAS has been questioned. However, the MTS and the DAROM rely on at least two different velocities of passive muscle stretching, and indicate only that the velocity for slow movement (V_1_) must be slower than the natural drop of the segment under gravity and that the velocity for fast movement (V_3_) must be less than gravity. Neither test provides measurable range of motion (ROM) or catch angle values or information on the corresponding velocities of these movements. Although goniometry provides measurement values of the ROM or catch angle, it does not permit simultaneous measurement of the corresponding passive movement angular velocity. Using an accelerometer attached to the DAROM enables the assessment of static contractures and dynamic spastic components with the following parameters: ROM deficit, catch angle, and corresponding angular velocities.

The other common method used to assess spasticity in the clinical setting is the pendulum test described by Wartenberg [10, 11]. The pendulum test is a biomechanical method that evaluates muscle tone using gravity to provoke the muscle stretch reflex during passive swinging of the lower leg. The Wartenberg test has been used in a variety of studies involving children with CP [10–17]. These studies suggest that the pendulum test may provide an objective measure that can be used to discriminate between various degrees of spasticity in children with CP. However, no information exists in the literature on whether the results of the pendulum test correlate with the results of other spasticity assessment methods in children with CP.

Currently, one of the most commonly used methods for assessing motor deficits in children with CP is instrumented gait analysis. However, most studies using gait analysis have evaluated a limited number of specific gait characteristics [3–5, 10, 11]. Because many gait parameters are interdependent, this approach can fail to account for the high degree of correlation among the various aspects of an individual’s gait.

To overcome these problems, the Gillette Gait Index (GGI; previously known as the Normalcy Index [NI]) [18], an index for quantifying the deviations from a normal gait, was introduced. The GGI was the first described index for quantifying deviations from normal gait, and has been validated in several patient populations [19–23]. It uses a single number to measure the deviation of a patient’s gait from the typical gait of a subject without any pathology. Importantly, the GGI values have been standardised within particular diagnostic categories in children with CP (types I, II, III, IV, hemiplegic, diplegic and quadriplegic) [18]. Additionally, the GGI has several attributes that make it well suited to comprehensive studies: it is robust, sensitive, reliable and objective [18, 19].

In our previous study [24], we found that deviations from normal gait (based on the GGI) in patients with CP generally do not depend on the static and/or dynamic contractures of the hip and knee flexors, as assessed using the DAROM. Therefore, one of the aims of this study was to examine the use of an accelerometer-based pendulum test to assess spasticity in children with CP via comparisons with other clinical measurements. Because the results of our previous study demonstrated that the DAROM in addition to the accelerometer-based system was a sensitive test of spasticity in children with CP, we decided to evaluate the utility of the pendulum test by comparing its outcomes with the results obtained using the DAROM test alone. However, our main purpose was to explore the relationship between the lower limb muscle spasticity as shown by pendulum test and deviations from normal gait in children with CP.

The present study was designed to verify the following hypotheses:

1. The pendulum test may provide an objective means of discriminating between typically developing children and children with CP and between various degrees of spasticity in the knee extensors in children with various types of CP, such as spastic hemiplegia (SH) and spastic diplegia (SD).
2. There is a good correlation between the degree of knee extensor spasticity measured using the DAROM and that measured using the pendulum test.
3. Deviations from normal gait in children with CP do not depend on the severity of knee extensor spasticity, as measured using the pendulum test.

## Methods

The study was approved by the Bioethical Committee of the Medical University of Silesia in Katowice, and the patients and their parents/guardians provided written informed consent prior to study enrolment and data collection.

### Subjects

A total of 36 children with CP, including 18 with SH and 18 with SD, were included in this study. All of the patients had a physician’s diagnosis of either SH or SD and were independently functioning outpatients (Level I or II on the Gross Motor Function Classification System) [25] at local paediatric rehabilitation centres. Additionally, they were clinically evaluated (using the DAROM) prior to this study, and all were found to have unilateral (SH) or bilateral (SD) spasticity.

All subjects met the following criteria: (1) age over 7 years (to minimise the incidence of unstable kinematic parameters), (2) the ability to follow verbal directions, (3) no history of surgical procedures on the lower extremities and (4) the ability to actively extend the knee from 90 to 45 degrees while in a sitting position. Additional criteria for the children with CP included the following: (1) a diagnosis of SH or SD, (2) the ability to actively extend the knee without simultaneous hip extension, (3) the ability to walk without assistance, (4) not taking any pharmacological agents at the time of the study and (5) no spasticity management for 6 months prior to the evaluation.

The SH group consisted of 6 girls and 12 boys; deficits occurred on the right side in 12 patients and on the left side in 6, and the mean age was 8 years, 2 months (range: 7 years, 4 months to 12 years, 2 months). The SD group consisted of 8 girls and 10 boys, and the mean age was 10 years and 4 months (range: 8 years, 3 months to 12 years, 2 months). A group of 18 typically developing children (6 girls and 12 boys), with a mean age of 8 years, 8 months (range: 7 years, 5 months to 12 years, 3 months), was recruited as a reference group (Ref). These children underwent only the pendulum test and three-dimensional gait analysis (3DGA).

### Testing procedure

**Figure 1:**
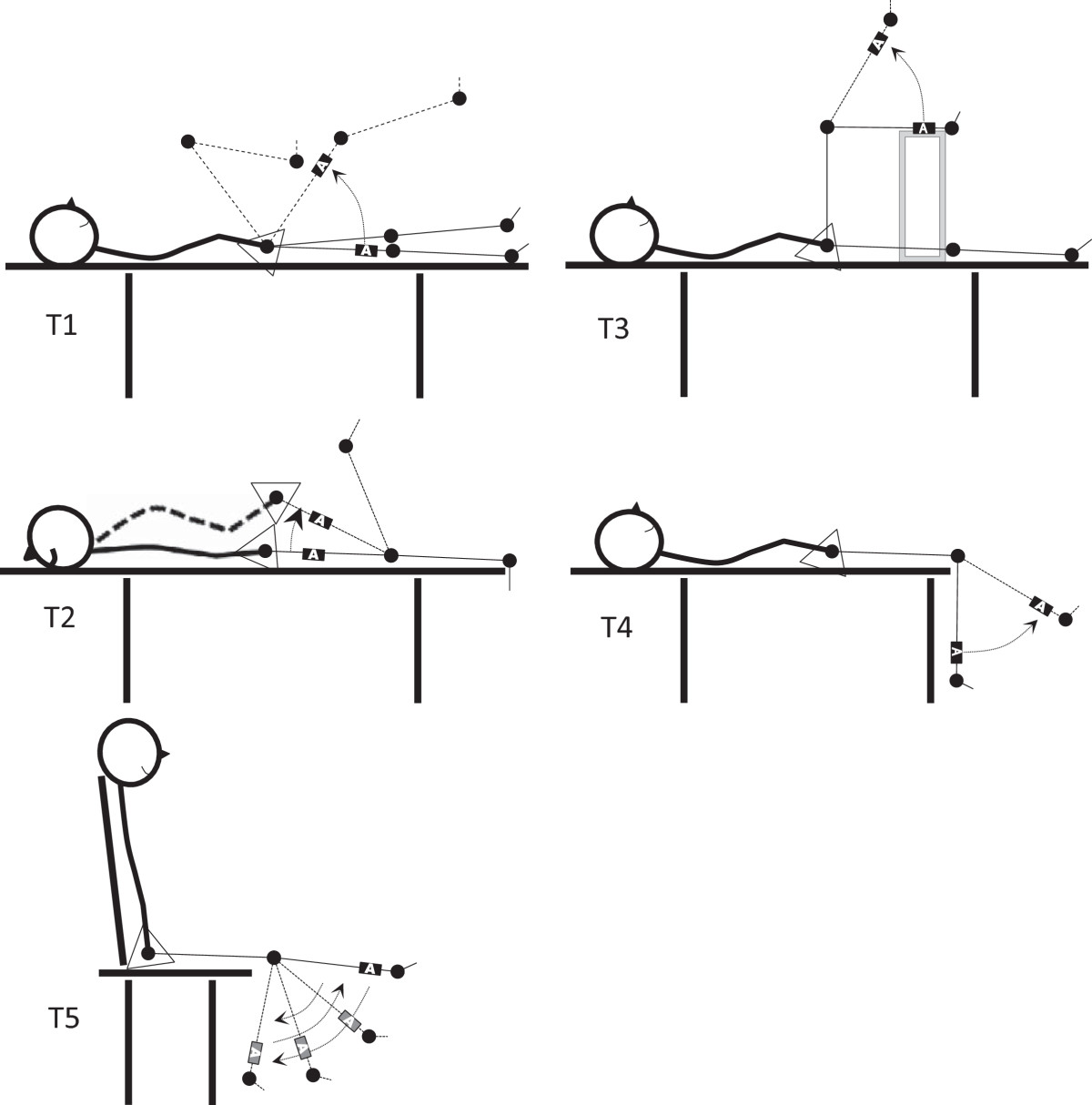
**Accelerometer placement and stabilisation for the dynamic assessment of the ROM (DAROM tests).** T1 – deficit of hip extension in the Thomas test, T2 – pelvis elevation angle in the Duncan–Ely test, T3 – popliteal angle, T4 – deficit of knee extension and T5 – pendulum test.

Each child with CP underwent multiple clinical measures of spasticity, including 4 DAROM tests and the pendulum test. Measurements were taken for both the left and right lower limbs. The testing positions and standardisation procedures for all measurements are shown in Figure 1. All measurements were performed at the Silesian Medical University Laboratory.For the pendulum test, each subject sat in a comfortable position on a specially designed couch with his or her trunk reclined approximately 20 degrees from vertical to minimise the effect of possible hamstring muscle tightness (seat-to-floor height of 50 cm), with the posterior calf not contacting the bench when the knee was in maximum flexion (Figure 1, T5). This movement was performed to ensure that the mat did not impede maximum knee flexion. The examiner positioned the subject’s leg to the point of a maximum passive knee extension and then released the leg to drop into flexion and continued to oscillate it with smaller excursions until the leg came to rest. Prior to the examination, the subject was instructed to let the leg swing freely once it was released by the examiner. The subjects’ relaxation during the pendulum tests was confirmed by an absence of visible movement or quadriceps femoris muscle contraction and free movements of the patella during passive medial–lateral mobilisation. Prior to data collection, 1 to 3 practice trials were performed. During data collection, the test was repeated if the examiner believed the subject was assisting or resisting the knee motions. The procedures were repeated until 3 trials (without interference) of each leg were obtained for each subject with approximately one minute between trials.

The between-trial reliability was calculated using intraclass correlation coefficients (ICC (3.3)) to measure the agreement. For the analysis, mean ICC values of 0.90 and higher indicated excellent reliability, values between 0.89 and 0.80 very good, values between 0.70 and 0.79 good, and those below 0.70 indicated moderate (0.40–0.69), fair (0.20–0.39) and poor (<0.20) reliability.

Every outcome from the pendulum test demonstrated high and very high reliability for both groups of subjects, with ICC (3.3) values ranging from 0.79 to 0.95 for the children with CP (95% confidence interval [CI], 0.73 to 0.96) and from 0.88 to 0.99 (95% CI, 0.77 to 0.99) for six variables among the typically developing children. The first swing excursion demonstrated an excellent repeatability of 0.99 (95% CI, 0.97 to 0.99) for the typically developing children and 0.95 (95% CI, 0.87 to 0.98) for the children with CP.

For DAROM tests 1–4, the testing procedure has been described in detail in our previous paper [24].

Joint motion during both the DAROM and the pendulum tests was measured by one examiner using an accelerometer-based system with ZK software (Institute of Electronics of Silesian University of Technology Poland) [24, 26]. The transducer of the accelerometer transmits a digitised time-dependent function of the angle between the patient’s thigh and leg axes via an interface using a special computer programme. The function is registered, and coefficients describing the extremity of motion in the joint are determined [27].

### Data collection and analysis

The outcome measures were collected from clinical measurements of spasticity: the pendulum test [15, 28] and DAROM tests. The results are presented in Table 1.

**Table 1:** **Definitions of the outcome measures collected from the clinical measurements of spasticity**

Range of motion deficit (DROM) for DAROM tests: T1, T2, T3, T4	**DROM I**	Range of motion deficit following a slow velocity stretch (V_1_); expressed in degrees.
	**DROM II**	Range of motion deficit after a fast velocity stretch (V_3_); expressed in degrees.
	**ASO**	Value was calculated as the difference between the DROM II and DROM I; expressed in degrees.
Outcome measures collected from the pendulum test: T5	**Ex**	First swing excursion. Defined as the difference between the starting angle (the position at which the examiner released the participant’s heel) and the first angle of reversal of the swinging limb; expressed in degrees.
	**RI**	Relaxation index. Calculated as follows: (starting angle – first angle)/(starting angle – resting angle), where the resting angle was the knee joint position maintained after oscillatory movement had ceased; expressed in degrees.
	**β**	Damping ratio. Defined as the ratio of the logarithmic decrement (δ) to the period; expressed in sec.
	**λ**	Defined as the natural log of the second to fourth peak amplitude ratio of the function φ (t).
	**t**	Duration of oscillations. Calculated as the duration of the pendulum swings (in seconds) from the time of lower limb release to the end of the final oscillation; expressed in sec.
	**n**	Determined by counting the number of sinusoidal waves produced by the swinging limb after the heel was released. The criterion for each oscillation was a flexion and extension wave with a minimum displacement toward the extension of a minimum of 3 degrees; number of oscillations.

### Three-dimensional instrumented gait analysis

After the clinical examination, an objective gait analysis was performed using the Compact Measuring System for 3D Real-Time Motion Analysis (CMS-HS 3D) with WinGait software (Zebris Medizintechnik GmbH, Germany). The CMS-HS 3D system is based on 15 active ultrasonic markers with 5 ultrasound marker triplets [29, 30]. Before the gait analysis, the following anatomical landmarks were identified using an instrumented pointer: the hip joint centre, knee rotation centre (internal and external), ankle rotation centre (internal and external), forefoot landmark (between the second and third metatarsals) and rear foot (heel). Gait data were recorded while the subjects walked on an Alfa XL treadmill (Kettler, Germany). Prior to data collection, all of the subjects were given the opportunity to practice walking on the treadmill. Each child’s normal overground walking speed, determined by measuring the time needed to travel a distance of 10 metres, was recorded by the same examiner before the gait analysis. According to the International Standards of Measurement, walking velocity is customarily expressed as meters per second. However, TM belt speeds are indicated in kilometres per hour. Therefore, based on the spontaneous walking speeds of the participants, the TM belt speeds were calculated in kilometres per hour. The children walked without shoes or assistive devices. Markers were attached to the skin with double-sided adhesive tape and placed bilaterally. Depending on each subject’s walking ability, 5 to 8 gait cycles were recorded. Three cycles (that did not include artefacts) were arbitrarily chosen for further analysis. The average data were then subjected to statistical analysis. The collected data were reported using WinGait software.

To characterise gait pattern, the GGI was calculated (separately for each lower limb) using the procedure described by Schutte [18]. The GGI is a single number derived from gait kinematics and spatiotemporal parameters that quantifies the deviation of a pathological gait from a normal gait. The GGI was based on 16 selected gait parameters taken from objective gait analysis data: time of toe off (% gait cycle), the walking speed/leg length, cadence (step/sec), mean pelvic tilt (°), range of pelvic tilt (°), mean pelvic rotation (°), minimum hip flexion (°), range of hip flexion (°), peak abduction in swing (°), mean hip rotation in stance (°), knee flexion at initial contact (°), time of peak knee flexion in the swing (% gait cycle), range of knee flexion (°), peak dorsiflexion in stance (°), peak dorsiflexion in swing (°) and mean foot progression (°).

### Statistical analysis

The mean values of the various parameters from the normal right and left lower limbs in the typically developing children, the unaffected and affected lower limbs in children with SH and the affected bilateral lower limbs in children with SD were calculated separately. The results of measurements from 3 trials each of the pendulum and DAROM tests were averaged.

One-way ANOVA was conducted between the groups to compare the effect of the level of involvement of the lower limbs on the scores from the pendulum test in children with SH, with SD and without CP (the Ref group). The following dependent variables were used: (1) (Ex) first swing excursion, (2) (RI) relaxation index, (3) (β) damping ratio, (4) (λ) logarithmic decrement, (5) (t) duration of oscillations and (6) (n) number of oscillations. An alpha level of 0.05 was chosen as a cutoff for all statistical comparisons, and a Bonferroni post hoc comparison was performed. The level of significance was set at *p* < 0.05.

The correlation coefficients between the DAROM results and the pendulum test outcomes for each type of lower limb in the children with CP were calculated using the Spearman rank correlation coefficient. To establish the dependence of GGI on the scores from the pendulum test, the Spearman rank correlation test was used.

The Statistica (version 10.0 PL) software package was used for statistical analysis.

## Results

### Clinical measurement data analysis

**Figure 2:**
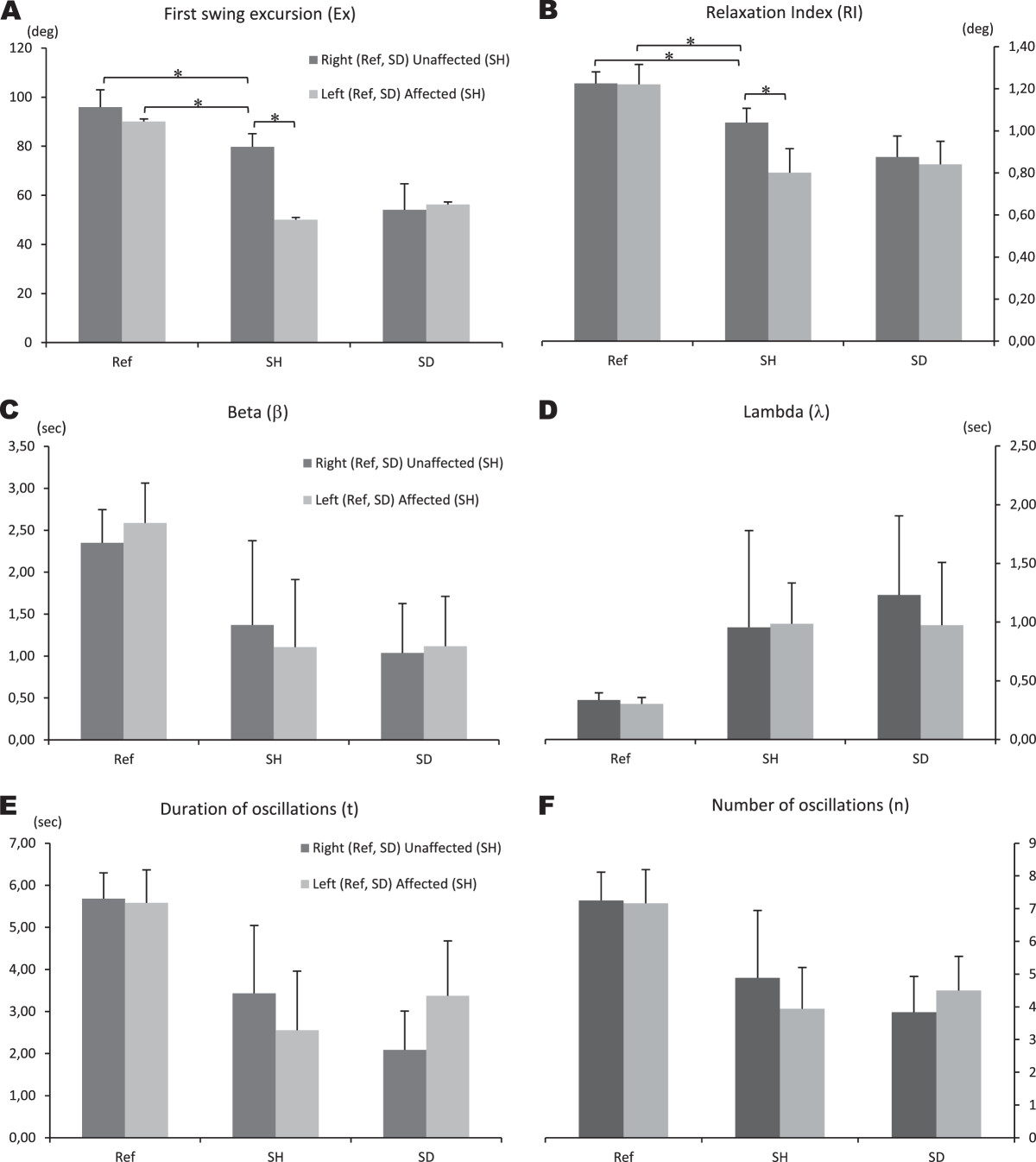
**Analysis of variance results.** Differences between accelerometer–based pendulum test variables: **A)** First swing excursion; **B)** Relaxation Index; **C)** Beta; **D)** Lambda; **E)** Duration of oscillations; **F)** Number of oscillations, within each type of lower limb: right and left lower limbs of controls (Ref), unaffected and affected lower limbs of children with spastic hemiplegia (SH) and bilateral affected lower limbs of children with spastic diplegia (SD).

On average, the children diagnosed with CP presented pendulum test results that were approximately two times worse than those of the children without CP (Figure 2). Additionally, the Ex and RI variables calculated for the unaffected lower limbs of the subjects with SH were significantly lower than those calculated for the affected hemiplegic and diplegic limbs but much higher than those for the normal lower limbs of the controls (each p-value < 0.001; Figure 2). On average, the Ex and RI variables were also lower for the unaffected lower limbs of the children with SH (80°) compared with those of typically developing children (95° and 91° for the right and left legs, respectively; p_1_ = 0.00012, p_2_ = 0.00014; Figure 2).

The differences among the normal limb movements of typically developing children, the movements of the unaffected and affected lower limbs in children with SH and the movements of the affected lower limbs in children with SD were compared using an ANOVA test and are presented in Figure 2. In general, there was a significant main effect of the level of involvement of the lower limbs on all parameters of the pendulum test: *FEX* (5.90) = 88.13, *p* < 0.001; *FRI* (5.90) = 58.46, *p* < 0.001; *F*β (5.90) = 13.17, *p* < 0.001; *F*λ (5.90) = 6.94, *p* < 0.001; *Ft* (5.90) = 21.94, *p* < 0.001; and F*n* (5.90) = 18.99, *p* < 0.001 (Figure 2).

The DAROM results for the unaffected and affected lower limbs of the children with SH and the affected lower limbs of those with SD have been described in detail in our previous paper [24].

### Correlations between the DAROM test (1–4) data and the scores of the accelerometer-based pendulum test

Correlations between the biomechanical parameters and the outcome measures from the pendulum test are shown in Table 2. We found only fair (0.21–0.40) and moderate correlations (0.41–0.60); none of the correlations were good or very good. The correlation coefficients for the whole group of children with CP were not significant, except those between the angle of spasticity (ASO) in test 1–3 and 2 of the pendulum test parameters (Ex and RI), which showed low to moderate (0.22–0.45) correlations (Table 2).

**Table 2:** **Correlations between the biomechanical parameters and the outcome measures from the pendulum test**

Groups		SH + SD		SH		SH unaffected		SH affected		SD	
Parameters		Ex	RI	Ex	RI	Ex	RI	Ex	RI	Ex	RI
T 1	DROM I	-	-	-	-	-	0.48*	-	-	-	-
	DROM II	-	-	-	-	-	-	-	-	-	-
	ASO	-0.45***	-0.38***	-0.67***	-0.52***	-	-	-	-	-	-
T 2	DROM I	-	-	-	-	-	-	-	-	-	-
	DROM II	-0.25*	-	-	-	-	-	-	-	-	0.34*
	ASO	-0.35**	-0.22*	-	-	-	-	-	-	-	-
T 3	DROM I	-	-	-	-	-	-	-	-	-	-
	DROM II	-0.60***	-0.51***	-0.71***	-0.76***	0.49*	-	-	-0.53*	-	-
	ASO	-0.35**	-0.28*	-0.66***	-0.71***	-	-	-	-	-	-
T 4	DROM I	-	-	-0.34*	-0.35*	-	-	-	-	-	-
	DROM II	-	-	-0.58***	-0.42**	-	0.59**	-	-	-	-
	ASO	-	-	-0.48**	-0.36*	-	0.56**	-	-	-	-

SH, spastic hemiplegia; SD, spastic diplegia; Ex, first swing excursion; RI, relaxation index; DAROM tests, (T1, T2, T3, T4); DROM I, range of motion deficit following a slow velocity; DROM II, range of motion deficit following a fast velocity; ASO, angle of spasticity; *, p < 0.05; **, p < 0.01; ***, p < 0.001.

### Gillette Gait Index (GGI) analysis

**Figure 3:**
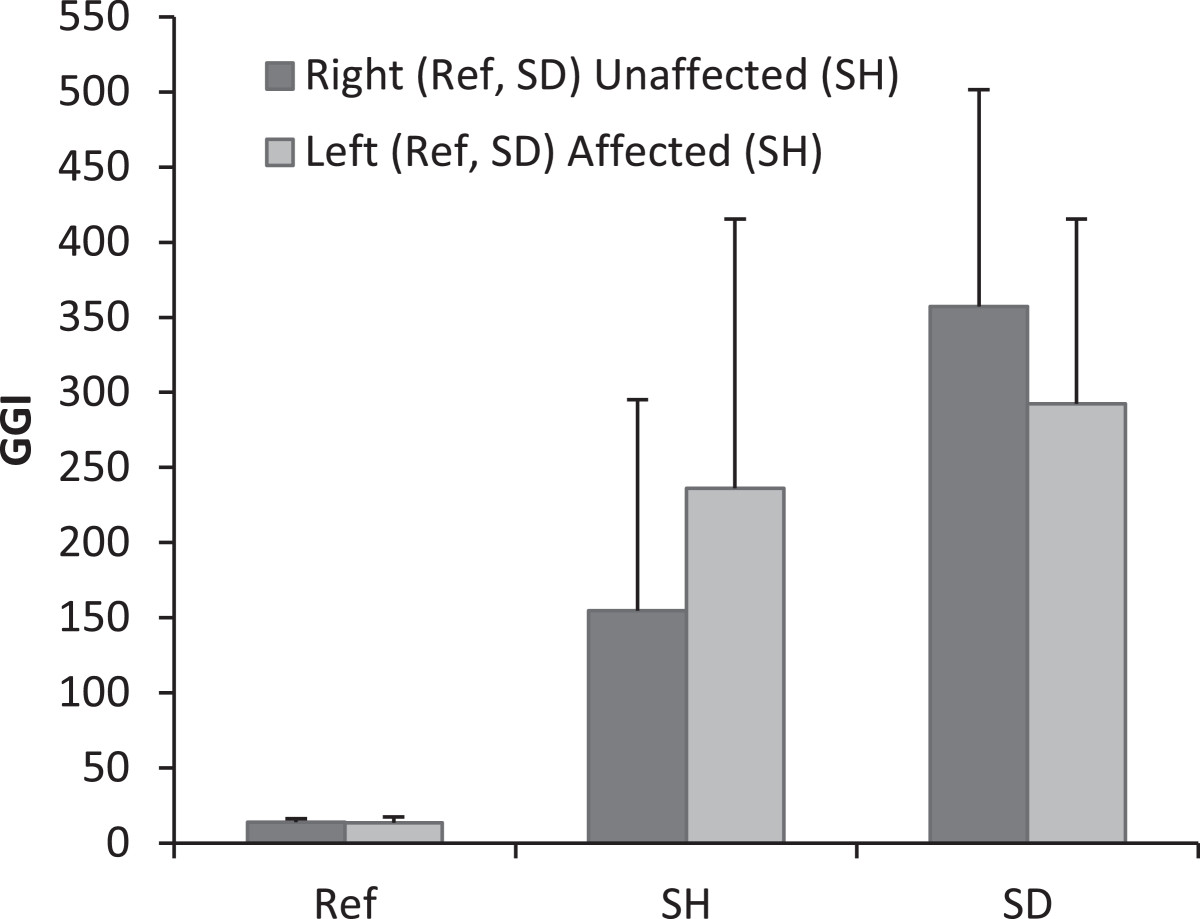
**Effect of the lower limb involvement level on the GGI.**

The GGI descriptive statistic for each lower limb of the typically developing children and for the children with SH and DS, as well as the dependence of the GGI on the level of lower limb involvement in children with CP, are shown in Figure 3. The indices of the typically developing children were all between 10 and 30. On average, the GGI values were highest for both diplegic lower limbs (292 for the left and 327 for the right), and the GGI value of the affected hemiplegic lower limb was higher than that of the unaffected one (226 and 154, respectively; Figure 3).The average and range of the GGI values could be used to distinguish unaffected, affected hemiplegic and affected diplegic lower limbs (each p-value < 0.001) in children with CP. In contrast, the GGI values could not be used to distinguish between the two diplegic lower limbs (Figure 3).

### Correlations between the pendulum test scores and the GGI

Generally, the correlation coefficients between the pendulum test scores and the GGI did not indicate significant correlations, except for measurements of the Ex, which showed a fair correlation (r_s_ = -0.316; Table 3).

**Table 3:** **Correlations between the pendulum test scores and the GGI values**

Test	Parameters	r_s_	p–value
T5	GGI & Ex	-0.316	**0.007**
	GGI & RI	-0.173	0.146
	GGI & β	0.002	0.984
	GGI & λ	0.133	0.265
	GGI & t	-0.013	0.916
	GGI & n	-0.014	0.909

T5, pendulum test; GGI, Gillette Gait Index; Ex, first swing excursion; RI, relaxation index; β, damping ratio; λ, logarithmic decrement; t, duration of oscillations; n, number of oscillations; r_s_, Spearman’s rank correlation; p–value, statistical significance. Significant correlations between clinical and GGI scores are indicated in bold.

## Discussion

Since Wartenberg's initial publication, electrogoniometry, videography and magnetic sensing devices [15–17, 31] have been used to characterise leg movements during oscillation after being dropped. The pendulum test was not used as a clinical tool to evaluate children with spastic cerebral palsy until the study by Fowler et al. [15], which raised the possibility that the pendulum test might provide a simple and objective means of quantifying spasticity [15]. One of the purposes of the present study was to verify the hypothesis that an accelerometer-based pendulum test might offer an objective means of discriminating between typically developing children and children with CP and among children with CP who present with various degrees of knee extensor spasticity. The obtained results revealed that all 6 variables calculated from the accelerometer-based pendulum test could be used to distinguish between the lower limbs of the control subjects and those of the children with CP. Additionally, two variables, Ex and RI, were sensitive enough that they could be used to differentiate the knee extensor spasticity between unaffected, affected hemiplegic and affected diplegic lower limbs in children with CP. The Ex variable quantified the effect of the severity of knee extensor spasticity and showed the most sensitivity to differences in muscle tone between the degree of lower limb spasticity in all participants. Although the hemiplegic subjects appeared to exhibit normal knee extensor tone in the unaffected lower limb, their Ex and RI values were significantly lower compared with those of typically developing children. This phenomenon was also described by Fowler et al. [12, 15], White et al. [16], Nordmark and Anderson [13] and Syczewska et al. [17].

One of the shortcomings of the present study was the reclining position of the patients during the pendulum test. This position does not allow for a deep stretch of the rectus femoris muscle, which could influence the spastic response during the test and may have affected the results. The semi-reclined trunk test position was used in the present study for two reasons. First, our initial goal was to perform these measurements in all children, even younger children and those with impaired cognitive function. The semi-reclined trunk test position offered the advantage that the children were comfortable and their tolerance of the test was very good. Because the pendulum test relies on the participants being relaxed and not assisting or resisting the pendular movements, we chose a test position in which the child was relaxed and safe, with one parent close behind. Second, most of the previous studies using the pendulum test to assess spasticity in patients with cerebral palsy were performed with the subjects in the half-lying position with the trunk inclined approximately 20°–40° from horizontal [13, 15, 32–34]. Performing the test with the trunk reclined to 20° allowed us to compare our findings with these previously reported studies. However, because there have been no prior studies investigating the influence of the semi-seated position in children with CP, we cannot entirely exclude the possibility that variations in the testing position could affect the results.

The DAROM combined with the accelerometer-based system is a sensitive test of spasticity in children with CP. It provides an objective value for both fixed contractures and the dynamic component of the ROM deficit, together with the angle of spasticity of the quadriceps and the hamstring muscles [24]. Repeating the DAROM analysis was not the aim of this study, but the correlation between the DAROM results and the outcomes of the pendulum test supports the validity of the pendulum test as tool for clinical spasticity measurement. Thus, we found that the angle of quadriceps spasticity (tests 1 and 2) and that of hamstring spasticity (test 3) mainly correlated with the Ex and the RI variables in the entire group of children with CP. Interestingly, in the SH group, this correlation was much stronger. In children with SD, the catch angle of the quadriceps (DROM II) correlated with other pendulum test parameters (the number and duration of oscillations and the logarithmic decrement and damping ratio). These findings suggest that there is a high level of variation in the scope of muscle contracture and the spasticity of the quadriceps in children with spastic CP.

To the best of our knowledge, the present study is the first to examine the use of accelerometer systems during pendulum tests of the lower limbs in children with CP. Our results confirmed the high sensitivity and excellent reliability of the pendulum test using an accelerometer-based system and suggest that this test provides an objective and reproducible measure of knee extensor spasticity in children diagnosed with CP. Importantly, the accelerometer system is less expensive than other movement analysis systems. Additionally, with this system, one device can be used to simultaneously perform multiple clinical measures of spasticity, including angular and linear parameters, and electrogoniometric measurements, which record only the angular parameter. These factors represent strengths of this study. The clinical examination evaluated isolated muscle groups, whereas pathological gait is defined by the interactions of multiple limitations, co-contractions and abnormal muscle synergies.

The pathological gait pattern in children with CP is characterised by compensation mechanisms [32, 35] to overcome the individual primary and secondary problems. The present study used the GGI to assess the individual gait pattern of each child with CP. The results demonstrated that, on average, a more severe diagnosis corresponds to more gait abnormalities and thus results in a higher GGI. The average GGI values and ranges obtained for both groups (typically developing children and children with CP) were similar to the research results for typically developing children and children diagnosed with type II and III hemiplegia and diplegia, as reported by Schutte et al. and Gage [18, 35]. The gait patterns of children with CP may not be “normal”, but they are nonetheless often functional in that they achieve the goal of locomotion through the use of available dynamic resources (including spasticity) [35]. The clinical examination focuses only on the primary and secondary problems of cerebral palsy and relies on the evaluation of isolated muscle groups. Thus, to accurately evaluate the extent of extent of deviations from normal gait, as well as the impact of muscle spasticity on these deviations, it is important to consider not only the degree of spasticity and particular gait parameters but also the relationship between these parameters. Measuring the relationship between spasticity and function is much more important and much more demanding than measuring spasticity alone. Therefore, the main purpose of this study was to explore the impact of knee extensor spasticity on the global index of deviations from a normal gait in children with CP.

The present findings indicate that there is only a limited relationship between the GGI values and the level of spasticity of the knee extensors. With the exception of the first excursion, there was no correlation between spasticity measurements derived from the accelerometer-based pendulum test. The correlation between the GGI and other spasticity measurement methods, such as the DAROM, which was discussed in detail in our previous paper [24], also suggest that deviations from a normal gait in patients with CP generally do not depend upon the static and/or dynamic contractures of the hip and knee muscles. Desloovere at al. [36] also reported only poor correlations between the degree of spasticity in the hip flexors and adductors and the quadriceps, hamstrings and gastrocnemius and particular gait parameters. Some clinicians, however, still believe the clinical examination provides sufficient information to define treatment in children with CP.

## Conclusions

Overall, this study demonstrates that the pendulum test, combined with an accelerometer-based system, may provide an objective method to differentiate between normal knee extensor muscles, and those with spasticity in children with CP, as well as between various degrees of spasticity, such as spastic hemiplegia and spastic diplegia. There was no correlation between the degree of spasticity in knee extensors in the DAROM analysis and the pendulum test. In addition, deviations from normal gait in children with CP were not correlated with the results of the pendulum test.

## Authors’ original submitted files for images

Below are the links to the authors’ original submitted files for images. Authors’ original file for figure 1 Authors’ original file for figure 2 Authors’ original file for figure 3

## Abbreviations

CP: Cerebral Palsy
SH: Spastic Hemiplegia
SD: Spastic Diplegia
Ref: Reference group
MAS: Modified Ashworth Scale
MTS: Modified Tardieu Scale
DAROM: Dynamic Evaluation of Range of Motion
ROM: range of motion
AOC: Angle of catch
DROM: Range of motion deficit, defined as the deficit value from the minimal muscle stretch position
DROM I: range of motion deficit following a slow velocity (V_1_) stretch
DROM II: Range of motion deficit that represents the angle of the catch after a fast velocity (V_3_) stretch
ASO: Angle of spasticity, which is the value calculated as the difference between the DROM II and DROM I
3DGA: three-dimensional gait analysis
GGI: Gillette Gait Index
NI: Normalcy Index
Ex: first swing excursion
RI: relaxation index
β: damping ratio
λ: logarithmic decrement
t: duration of oscillations
n: Number of oscillations
ANOVA: Analysis of variance
ICC (3.3): intraclass correlation coefficient
r_s_: Spearman’s rank correlation.
