# Supplementary figures and images for: Quadriceps femoris spasticity in children with cerebral palsy: measurement with the pendulum test and relationship with gait abnormalities
Source: J Neuroeng Rehabil. 2014 Dec 16;11:166. doi: 10.1186/1743-0003-11-166 (PMC4277843; doi:10.1186/1743-0003-11-166)

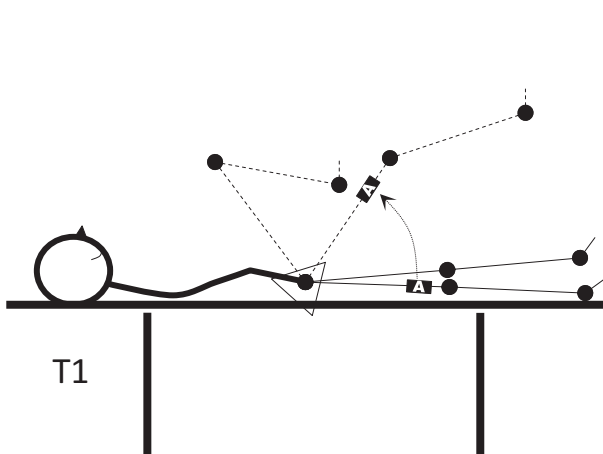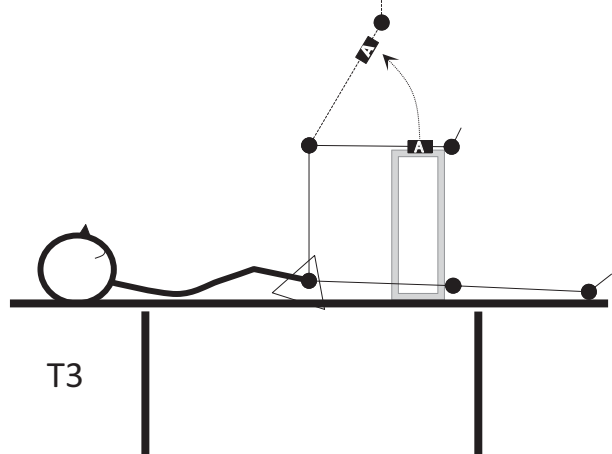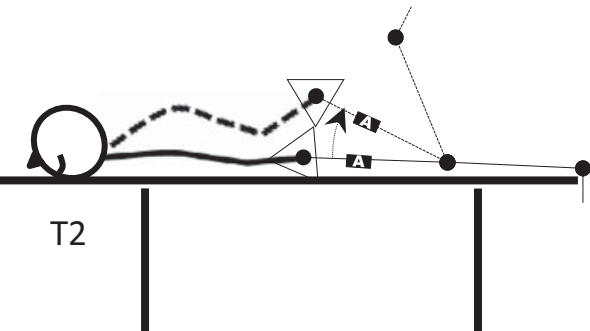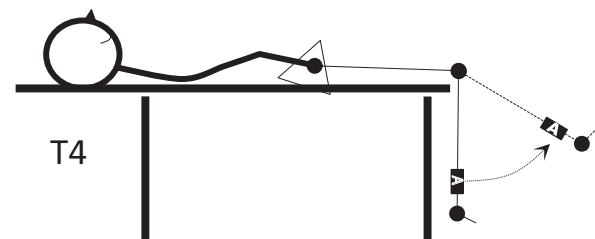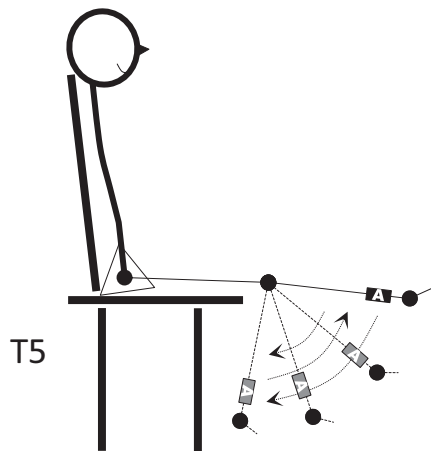

Supplement: Supplementary file 1 — Authors’ original file for figure 1 [file 12984_2013_685_MOESM1_ESM.pdf]

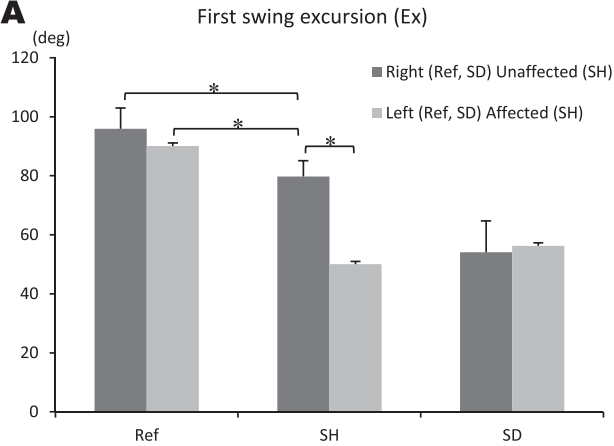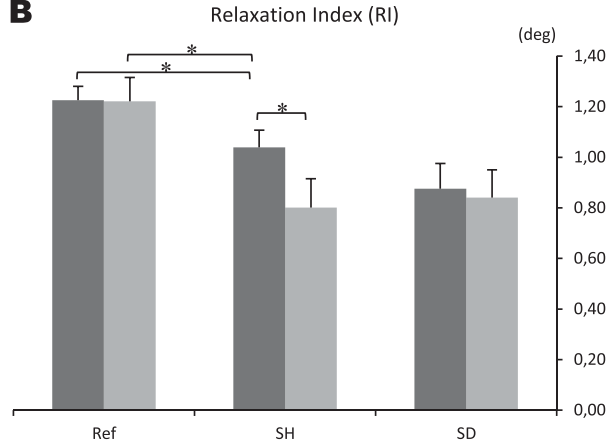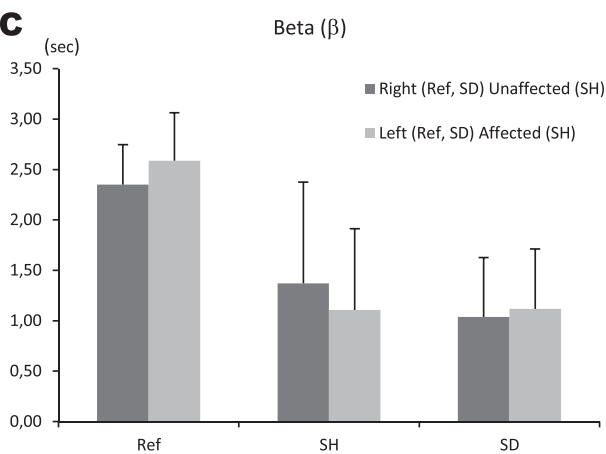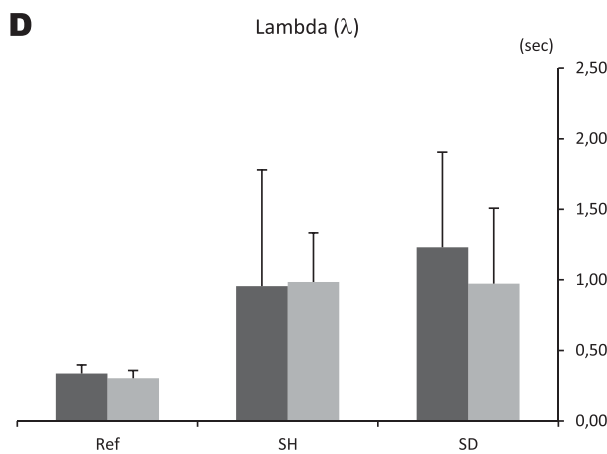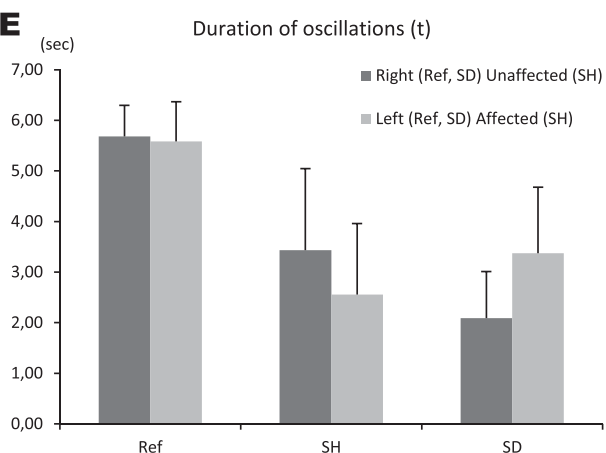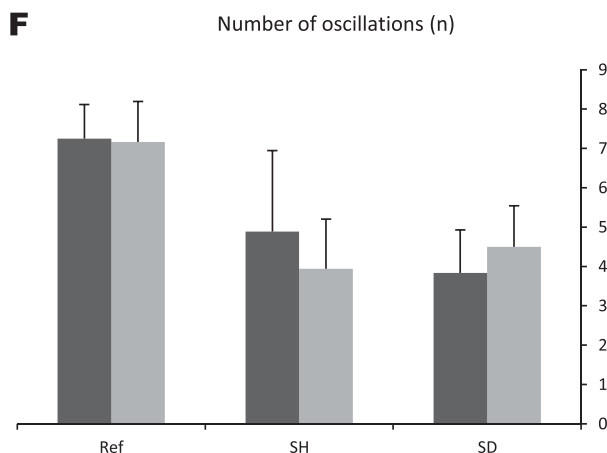

Supplement: Supplementary file 2 — Authors’ original file for figure 2 [file 12984_2013_685_MOESM2_ESM.pdf]

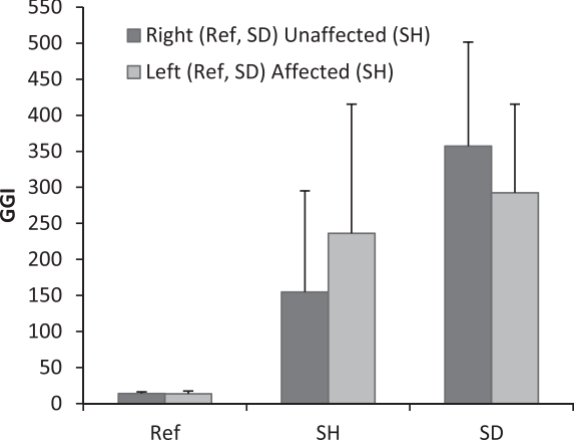

Supplement: Supplementary file 3 — Authors’ original file for figure 3 [file 12984_2013_685_MOESM3_ESM.pdf]
